# *PRNP *promoter polymorphisms are associated with BSE susceptibility in Swiss and German cattle

**DOI:** 10.1186/1471-2156-8-15

**Published:** 2007-04-16

**Authors:** Bianca Haase, Marcus G Doherr, Torsten Seuberlich, Cord Drögemüller, Gaudenz Dolf, Petra Nicken, Katrin Schiebel, Ute Ziegler, Martin H Groschup, Andreas Zurbriggen, Tosso Leeb

**Affiliations:** 1 Institute of Genetics, Vetsuisse Faculty, University of Berne, Bremgartenstr. 109a, 3001 Berne, Switzerland; 2 Division of Clinical Research, Dept. Clinical Veterinary Medicine, Vetsuisse Faculty, University of Berne, 3001 Berne, Switzerland; 3 NeuroCenter, Reference Laboratory for TSE in animals, Vetsuisse Faculty, University of Berne, 3001 Berne, Switzerland; 4 Center for Food Science, Food Toxicology, University of Veterinary Medicine Hannover, Bischofsholer Damm 15, 30173 Hannover, Germany; 5 Institute for Biochemistry, Emil-Fischer-Center, University of Erlangen-Nürnberg, Fahrstr. 17, 91054 Erlangen, Germany; 6 Friedrich-Loeffler-Institut (FLI), Institute for Novel and Emerging Infectious Diseases, Boddenblick 5a, 17493 Greifswald – Insel Riems, Germany

## Abstract

**Background:**

Non-synonymous polymorphisms within the prion protein gene (*PRNP*) influence the susceptibility and incubation time for transmissible spongiform encephalopathies (TSE) in some species such as sheep and humans. In cattle, none of the known polymorphisms within the *PRNP *coding region has a major influence on susceptibility to bovine spongiform encephalopathy (BSE). Recently, however, we demonstrated an association between susceptibility to BSE and a 23 bp insertion/deletion (indel) polymorphism and a 12 bp indel polymorphism within the putative *PRNP *promoter region using 43 German BSE cases and 48 German control cattle. The objective of this study was to extend this work by including a larger number of BSE cases and control cattle of German and Swiss origin.

**Results:**

Allele, genotype and haplotype frequencies of the two indel polymorphisms were determined in 449 BSE cattle and 431 unaffected cattle from Switzerland and Germany including all 43 German BSE and 16 German control animals from the original study. When breeds with similar allele and genotype distributions were compared, the 23 bp indel polymorphism again showed a significant association with susceptibility to BSE. However, some additional breed-specific allele and genotype distributions were identified, mainly related to the Brown breeds.

**Conclusion:**

Our study corroborated earlier findings that polymorphisms in the *PRNP *promoter region have an influence on susceptibility to BSE. However, breed-specific differences exist that need to be accounted for when analyzing such data.

## Background

The incidence of transmissible spongiform encephalopathies (TSE), also referred to as prion diseases, in a defined host population is influenced by a variety of factors. In the individual animal, however, the disease is always associated with an increase of the protease-resistant form of the cellular prion protein, which is then denoted scrapie-associated prion protein (PrP^sc^) [1]. The ingestion of meat and bone meal (MBM) produced from scrapie-infected sheep or from cattle with BSE represents the most likely cause of the large BSE outbreak in cattle in the United Kingdom [2]. The function of the physiological prion protein isoform (PrP^c^) has not yet been elucidated. It has been proposed that PrP^c ^plays a role in normal synaptic function [3] or in cell-cell interactions and acts as an anti-apoptotic signaling molecule [4].

In infectious TSEs, the exact route of propagation of the infectious agent is still under dispute. However, it is generally accepted that the gastrointestinal tract plays an important part in the pathogenesis. Prions are probably absorbed by the gut and transported to the brain, where they cause neurodegeneration and aggregation of insoluble PrP^Sc^. Although there is no primary immunological response to the infectious agent, there is an ongoing debate about the involvement of the lymphoreticular system in this propagation [5-10].

The modulation of susceptibility to prion diseases by genetic factors was initially discovered in sheep, and is now known from various species. Several mutations within the coding region of the prion protein gene (*PRNP*) modulate scrapie susceptibility in sheep [11-15]. A similar situation exists in humans where a polymorphism at codon 129 of the *PRNP *coding region is highly correlated with susceptibility to variant Creutzfeldt-Jakob (vCJD) disease, the human infectious TSE that originates from exposure to cattle BSE. All human vCJD patients share the homozygous ^129^Met/^129^Met genotype, whereas no homozygous ^129^Val/^129^Val or heterozygous vCJD patients have been diagnosed to date with the exception of some iatrogenic cases that were caused by blood transfusions in pre-symptomatic vCJD patients. In cattle, however, none of the known polymorphisms within the *PRNP *coding region seems to have an influence on susceptibility to BSE.

Early on, the hypothesis was formulated that changes in expression of the endogenous PrP^c ^might influence susceptibility to TSE and/or TSE incubation time [16]. Consistent with this hypothesis, the level of PrP^c ^expression in transgenic mice is inversely correlated with the incubation time for TSE [17]. In a previous study we demonstrated the first tentative association of susceptibility to BSE with polymorphisms in the promoter region of the bovine *PRNP *gene [18]. The allele frequencies of a 23 bp indel polymorphism at position 1594 relative to the transcription start site and a 12 bp indel polymorphism at position +300 in the first intron showed significant associations with BSE. The most common haplotypes of these polymorphisms were either insertion-insertion (referred to as I-I) or deletion-deletion (referred to as D-D). Functional promoter studies indicated that the 23 bp insertion allele is able to bind the repressing RP-58 transcription factor, which mediates its repressing effects via interaction with specificity protein 1 (SP1) [19]. The 12 bp insertion allele represents a functional SP1 binding site. From the available evidence a model was proposed: In the I-I haplotype, RP-58 binds to the 23 bp insertion and exerts a repressing effect via SP1, which is bound at the 12 bp insertion. In the D-D haplotype this repression cannot take place and the resulting *PRNP *expression is higher than in the I-I haplotype [20]. The effect of the 12 bp indel polymorphism was recently confirmed in independent studies using German and English cattle [21-23].

From studies in mice it is known that other genetic factors unlinked to the *PRNP *gene can also influence TSE susceptibility. Two genome-wide searches for other TSE susceptibility genes in cattle have been conducted. A genome-wide association analysis with microsatellite markers and transmission disequilibrium tests (TDT) revealed associations between markers on chromosomes 5, 10 and 20 and BSE infection [24]. A QTL search using half-sib families resulted in QTL regions for BSE that were located in different regions of the bovine genome on BTA 17 and BTA X/Y [25].

In this study, we compared the allele and genotype frequencies of the 23 bp and 12 bp indel *PRNP *promoter polymorphisms between 449 BSE affected cattle and 431 unaffected controls to confirm the earlier-reported association in a large sample. Our sample consisted of German and Swiss cattle. Up to 2006 there were only 405 confirmed cases of BSE in Germany, therefore some overlap of our German BSE cohort with the BSE cohorts of previous studies was inevitable. However, we used a different German control cohort and we also investigated Swiss cattle for the first time. Swiss cattle seemed to be suitable for extending the available animal material as some breeds within Germany and Switzerland are related. Furthermore, a sufficient number of confirmed BSE cases was available in Switzerland.

## Results

### *PRNP *genotypes and association to BSE susceptibility

A total of 880 cattle from Switzerland and Germany were available for this study. The animals included 449 BSE-affected animals (245 Swiss, 204 German) and 431 control animals (250 Swiss, 181 German). For the Swiss samples the group of control animals matched the BSE group in respect of sex, breed and age. The German control group was matched to the German BSE cases in breed structure (frequency matching). Owing to very stringent data protection measures in Germany, matching of cases and controls by age and sex was not possible.

DNA samples from all animals were genotyped at the 23 bp indel polymorphism and the 12 bp indel polymorphism within the bovine *PRNP *promoter region and intron 1. For each of the two polymorphisms the distribution of alleles and genotypes between BSE-affected and healthy cattle was investigated separately within each breed. Both markers were in Hardy-Weinberg equilibrium in all breeds. Haplotypes consisting of the two indel alleles were assigned to the animals. There was extensive linkage disequilibrium between the two markers (r^2 ^= 0.63). In agreement with our previous study [18] all genotypes could be explained by the three haplotypes I-I, D-I and D-D.

It turned out that animals from the Brown breeds (98 Swiss Brown BSE animals, 103 Swiss Brown controls, 16 German Brown BSE animals and 41 German Brown controls) had significantly different allele frequencies at the 23 bp and 12 bp indel polymorphisms from all other breeds, while animals from all other breeds (Swiss Schwarzfleck, Swiss Simmental × Red Holstein, German Fleckvieh, German Holstein) showed comparable allele frequencies. In animals from the Brown breeds the insertion alleles at both polymorphisms occurred with higher frequency than in the other breeds.

On the basis of their similar allele frequencies in the control groups, the latter breeds were pooled and the association between the two investigated polymorphisms and BSE status was analyzed using that pool. Statistical analysis of the allele frequencies across the pooled group demonstrated that the allele and genotype distribution of the 23 bp polymorphism is significantly associated with BSE infection (*P *< 0.05; Table 1). No significant association could be detected for the 12 bp indel alone; however, the haplotypes consisting of the alleles at the 23 bp indel and the 12 bp indel again showed a significant association with BSE. At the 23 bp indel polymorphism the deletion alleles were overrepresented in the BSE group.

**Table 1:** Allele, genotype, and haplotype frequencies of BSE-affected cattle and controls

**Pooled breeds from Germany and Switzerland**: **German Holstein, German Fleckvieh, Swiss Schwarzfleck, Swiss Simmental × Red Holstein,**										
										
		Allele frequency				Genotype frequency				
				
**23 bp indel**	n	D	I	*P*		D/D	D/I	I/I	*P*	HWE_pval_
Total	1244	0.65	0.36	**0.0105**		0.41	0.47	0.12	**0.0207**	0.6559
Control	574	0.61	0.39			0.37	0.47	0.16		
BSE	670	0.68	0.32			0.45	0.47	0.09		
										
		Allele frequency				Genotype frequency				
				
**12 bp indel**	n	D	I	*P*		D/D	D/I	I/I	*P*	HWE_pval_

Total	1244	0.56	0.44	0.1209		0.33	0.47	0.20	0.1731	0.3931
Control	574	0.54	0.46			0.31	0.46	0.23		
BSE	670	0.58	0.42			0.34	0.49	0.17		
										
**23 & 12 bp indel**		Haplotype frequency								
							
	n	D-D	D-I	I-I	*P*					

Total	1244	0.56	0.09	0.36	**0.0169**					
Control	574	0.54	0.07	0.39						
BSE	670	0.58	0.10	0.32						
										
**Pooled breeds from Switzerland**: **Swiss Schwarzfleck, Swiss Simmental × Red Holstein**										
										
		Allele frequency				Genotype frequency				
				
**23 bp indel**	n	D	I	*P*		D/D	D/I	I/I	*P*	HWE_pval_

Total	588	0.59	0.42	**0.0295**		0.33	0.50	0.16	0.0614	0.6306
Control	294	0.54	0.46			0.29	0.50	0.21		
BSE	294	0.63	0.37			0.37	0.51	0.12		
										
		Allele frequency				Genotype frequency				
				
**12 bp indel**	n	D	I	*P*		D/D	D/I	I/I	*P*	HWE_pval_
Total	588	0.48	0.52	0.4091		0.24	0.48	0.28	0.5795	0.6396
Control	294	0.46	0.54			0.23	0.46	0.31		
BSE	294	0.50	0.50			0.25	0.50	0.25		
										
**23 & 12 bp indel**		Haplotype frequency								
							
	n	D-D	D-I	I-I	*P*					
Total	588	0.48	0.11	0.42	**0.0218**					
Control	294	0.46	0.08	0.46						
BSE	294	0.49	0.14	0.37						
										
**Pooled Brown breeds from Germany and Switzerland**: **German Brown, Swiss Brown**										
										
		Allele frequency				Genotype frequency				
				
**23 bp indel**	n	D	I	*P*		D/D	D/I	I/I	*P*	HWE_pval_
Total	516	0.42	0.59	0.5357		0.20	0.43	0.37	0.3574	0.0630
Control	288	0.40	0.60			0.17	0.46	0.37		
BSE	228	0.43	0.57			0.24	0.39	0.38		
										
		Allele frequency				Genotype frequency				
				
**12 bp indel**	n	D	I	*P*		D/D	D/I	I/I	*P*	HWE_pval_
Total	516	0.23	0.78	0.1432		0.04	0.38	0.58	0.1317	0.4117
Control	288	0.21	0.79			0.02	0.38	0.60		
BSE	228	0.26	0.74			0.07	0.39	0.54		
										
**23 & 12 bp indel**		Haplotype frequency								
							
	n	D-D	D-I	I-I	*P*					
Total	516	0.23	0.19	0.58	0.2439					
Control	288	0.21	0.20	0.59						
BSE	228	0.26	0.17	0.57						

In addition, we separately analyzed the Swiss cattle as in this population *PRNP *polymorphisms and their association with BSE have not previously been investigated. Within the pooled Swiss breed group (Schwarzfleck, Simmental × Red Holstein) only the allele frequency but not the genotype frequency at the 23 bp indel was significantly associated with BSE (*P *< 0.05; Table 1). Again the deletion allele at the 23 bp indel polymorphism was more frequently found in the BSE-affected group. This trend could also be seen in the genotype frequencies of the 23 bp indel polymorphism although it was not statistically significant.

Animals of the Brown breeds (Swiss Brown, German Brown) were analyzed separately in view of the aforementioned differences from the other breeds in their allele frequency distributions at the two polymorphisms studied. Neither the 23 bp indel polymorphism nor the 12 bp indel polymorphism was significantly associated with BSE status (Table 1).

### Risk factor assessment

We estimated the magnitude and direction of the association between *PRNP *indel polymorphisms and BSE using multivariable logistic regression and odds ratios. As all previous studies had shown that the deletion alleles at the 23 bp and 12 bp indel polymorphisms are overrepresented in BSE affected animals we used those animals carrying the potentially most resistant diplotype, I-I/I-I, as (baseline) comparison group. The logistic regression model contained BSE status as the outcome, diplotype as risk factor, and country of origin (Switzerland, Germany) in order to control for the potentially confounding effect. Again, all cattle of the Brown breeds were analyzed separately.

The odds ratio for animals of the pooled breed group carrying the D-D/D-I diplotype to develop BSE was 2.49 ± 0.37 (mean ± standard error) compared to animals with the I-I/I-I diplotype (*P *= 0.01). The odds ratio for animals with the D-D/D-D diplotype to develop BSE was 1.76 ± 0.28 (*P *= 0.04), and for animals with the I-I/D-D diplotype 1.66 ± 0.27 (*P *= 0.05). The D-I/D-I diplotype was disregarded because of its low frequency within the pooled group. For Swiss and German Brown no statistically significant higher risks could be observed for specific genotypes.

## Discussion

In a previous study of the bovine *PRNP *gene we investigated the promoter region of this gene to search for polymorphisms that affect susceptibility to BSE [18]. We and others hypothesized that mutations within this region might influence the level of *PRNP *expression and consequently might have an impact on susceptibility to BSE. An association of BSE susceptibility with respect to *PRNP *genotypes at the 23 bp indel polymorphism in the 5'flanking region and the 12 bp indel polymorphism within intron 1 of the *PRNP *gene was demonstrated in a small sample of animals. Recently, similar associations were confirmed in larger replicating association studies using German and British cattle [21-23]. It has to be mentioned that the design of the previous and the present association studies does not allow to determine whether a difference between the genotype frequencies of BSE-affected animals and controls is due to a genetic effect on susceptibility to BSE or to differences in the BSE incubation time. As it is not known whether some of the control cattle may have developed BSE later in life, effects on susceptibility to BSE or incubation time cannot be discriminated in these studies.

We have now validated the previous findings at the two indel polymorphisms in an enlarged sample consisting of both German and Swiss cattle; the latter were studied for the first time in regard to *PRNP *promoter polymorphisms. Differences between breeds were observed. Our findings clearly confirmed the previously-identified association of the deletion allele at the 23 bp indel polymorphism with BSE in a pooled sample of Swiss Schwarzfleck, Swiss Simmental × Red Holstein, German Fleckvieh and German Holstein cattle. We pooled these breeds as they had comparable allele frequencies at the markers studied [see Additional files 1, 2, 3]. The association could not be detected in the Brown breeds, which have a significantly higher frequency of the potentially more "resistant" insertion allele than all the other breeds studied. The paradoxical finding that Brown cattle have a high prevalence of BSE, although they also have a high frequency of the protective *PRNP *promoter genotypes, highlights the fact that BSE is an infectious disease, which is primarily influenced by environmental factors such as exposure to the infectious agent in the feed. The *PRNP *promoter genotype modulates disease susceptibility but obviously cannot confer absolute resistance on the animals.

The association of promoter polymorphisms has now been found several times in German cattle of different breeds [[18,21-23], this study], in the British Holstein population [22], and in Swiss cattle (this study). We and the investigators in all the previous German studies used control animals from different farms from the BSE-affected cattle. Obviously, matching the farms would have been preferable in order to match the exposure of the cases and controls to infectious prions. However, in all studies, comparable allele frequencies and also consistent associations at least in the trend were obtained with different sets of control animals. Therefore, the chosen sets of control animals seem to be truly representative of the breeds studied and the combined data from the studies indicate that the statistical associations are biologically significant.

There are, however, differences among the different studies in the answer to the question which specific polymorphism within the *PRNP *promoter shows the stronger association. In our previous study and in the present study we found the stronger effect at the 23 bp indel polymorphism and the weaker effect at the 12 bp indel polymorphism. Other studies found a stronger association with the 12 bp indel polymorphism [22] or did not analyze both polymorphisms together [21]. It will be very difficult to narrow down the list of potentially causative polymorphisms within the promoter region by association analyses as there is a high degree of linkage disequilibrium in the 5'-part of the *PRNP *gene [26]. To unravel the individual influence of each of the many existing promoter polymorphisms, functional assays for these different polymorphisms will be necessary to determine their respective contributions to the regulation of *PRNP *gene expression.

The size of the effect on susceptibility to BSE of the different *PRNP *genotypes was estimated by a risk factor assessment. We found a 1.76 times elevated odds ratio to develop BSE for animals carrying the diplotype D-D/D-D compared to animals with the diplotype I-I/I-I. This is consistent with the trend although smaller in magnitude than the odds ratio of 2.86 for the same diplotypes previously described for German and British cattle [22]. The relative risk or odds ratio of the potentially most deleterious diplotype D-I/D-I cannot be estimated with high accuracy as the frequency of this diplotype is very low in the cattle populations studied.

## Conclusion

The association between promoter polymorphisms within the bovine *PRNP *gene and susceptibility to BSE could be confirmed in a large sample across multiple breeds from Germany and Switzerland. Breed-specific differences exist and the association seems to be strong in Holstein- and Simmental-related breeds, whereas it is not detectable in the Brown breeds. The deletion alleles at the 23 bp and 12 bp indel polymorphisms confer a higher risk of developing BSE on the breeds studied.

## Methods

### DNA samples

DNA was isolated from blood or tissue homogenates from different cattle breeds using the Nucleon BACC2 kit (Amersham Biosciences) according to the manufacturer's protocol. For decontamination, two phenol-chloroform extractions were included. Altogether, 449 BSE-affected cattle and a control group of 431 unaffected animals were analyzed. The BSE-affected group is based on 245 samples from Switzerland and 204 samples from Germany [see Additional file 4]. For the Swiss animals, data about breed and age were available, whereas no data were available for the German group at the beginning of this study. The composition of the Swiss control group was based on age, sex, and breed structure of the affected group; the German control group was arranged with respect to the breed structure of the German BSE-affected group after data about this group became available.

### Genotyping

PCRs flanking the 23 indel polymorphism (AJ298878.1:g.47836_47837ins23) and the 12 bp indel polymorphism (AJ298878.1:g.49729_47730ins12) were carried out and the product sizes were evaluated on agarose gels as described previously [18]. PCR was carried out in a 52 μl reaction volume containing 20 ng DNA, 1 unit AmpliTaq Gold (Applied Biosystems, Rotkreuz, Switzerland), 10 pmol of each primer, 5 mM dNTPs (Roth) and 1.5 mM MgCl_2 _in the buffer supplied by the manufacturer. The amplification was performed using an initial denaturation at 95°C for 15 min, followed by 40 cycles of denaturation at 94°C for 30 s, annealing at 56°C for 30 s and extension at 72°C for 30 s. Finally an extension step at 72°C for 5 min was performed.

### Haplotype estimation, Hardy-Weinberg equilibrium and linkage disequilibrium

Initially, haplotypes were estimated subjectively from the unphased genotypes without the help of any computer software. The haplotype estimation was based on the assumption that out of the four theoretically possible haplotypes, only D-D, D-I and I-I exist in the cattle populations studied. The haplotypes were also estimated from the unphased genotypes with the program PHASE 5.1 [27]. PHASE predicted exactly the same haplotypes for all 880 animals as the subjective haplotype estimation. Hardy-Weinberg equilibrium and linkage disequilibrium were calculated with the program Haploview 3.32 [28].

### Statistical analyses

Allele, genotype and haplotype frequencies, as well as the risk factor assessment for association with BSE infections, were calculated using NCSS 2004 statistical software [29]. The association between allele, genotype and haplotype frequencies and breed as well as BSE status was analyzed using cross-tabulation with Chi-square (for 2 × 2 tables) and Fisher's exact tests. Risk factor assessment was performed by a multivariable logistic regression model containing BSE status (yes/no) as the outcome, genotype (4 distinct levels) as risk factor, and country of origin (Switzerland, Germany) in order to control for the potentially confounding effect. After exploring the allele and genotype frequencies separately for the control groups of each breed, we decided to pool those breeds with similar allele and genotype frequencies, and to conduct further analyses on those breeds together.

## Authors' contributions

BH performed the majority of the genotyping experiments and wrote the initial draft of the manuscript. MD & BH, assisted by GD, performed the statistical analyses. TS and AZ provided the Swiss BSE samples and helped with the DNA isolation from infectious materials. CD and PN provided some genotypes. KS also provided some genotypes and reanalyzed some animals independently to check for genotyping errors. UZ and MHG provided the German BSE samples. TL provided the conceptual framework for the study and finalized the manuscript. All authors read and approved the final manuscript.

## Supplementary Material

Additional file 1 Allele frequencies within individual breeds. The table provided lists the allele frequencies for each breed separately. Click here for file

Additional file 2 Genotype frequencies within individual breeds. The table provided lists the genotype frequencies for each breed separately. Click here for file

Additional file 3 Haplotype frequencies within individual breeds. The table provided lists the haplotype frequencies for each breed separately. Click here for file

Additional file 4 Animals used in this study. The table provided lists the numbers of BSE and control animals for each breed separately. Click here for file
