# Supplementary material for: PRNP promoter polymorphisms are associated with BSE susceptibility in Swiss and German cattle
Source: BMC Genet. 2007 Apr 16;8:15. doi: 10.1186/1471-2156-8-15 (PMC1857697; doi:10.1186/1471-2156-8-15)
Supplement: Additional file 1 — Allele frequencies within individual breeds. The table provided lists the allele frequencies for each breed separately. [file 1471-2156-8-15-S1.doc]

## Additional File 1 - Allele frequencies within individual breeds

|  | Frequency of deletion | | | |  |
| --- | --- | --- | --- | --- | --- |
| **23 bp indel** | BSE | n | Control | n | P |
| German Holstein | 0.681 | 238 | 0.669 | 160 | 0.8032 |
| German Fleckvieh | 0.775 | 138 | 0.692 | 120 | 0.1280 |
| German Brown | 0.531 | 32 | 0.439 | 82 | 0.3750 |
| Swiss Brown | 0.413 | 196 | 0.388 | 206 | 0.6103 |
| Swiss Schwarzfleck | 0.640 | 50 | 0.481 | 52 | 0.1054 |
| Swiss Simmental x Red Holstein | 0.627 | 244 | 0.554 | 242 | 0.1002 |
|  |  |  |  |  |  |
| **12 bp indel** |  |  |  |  |  |
| German Holstein | 0.609 | 238 | 0.613 | 160 | 0.9479 |
| German Fleckvieh | 0.569 | 138 | 0.431 | 120 | 0.1141 |
| German Brown | 0.281 | 32 | 0.159 | 82 | 0.1357 |
| Swiss Brown | 0.228 | 196 | 0.260 | 206 | 0.4544 |
| Swiss Schwarzfleck | 0.560 | 50 | 0.423 | 52 | 0.1667 |
| Swiss Simmental x Red Holstein | 0.484 | 244 | 0.471 | 242 | 0.7821 |
