# Supplementary material for: PRNP promoter polymorphisms are associated with BSE susceptibility in Swiss and German cattle
Source: BMC Genet. 2007 Apr 16;8:15. doi: 10.1186/1471-2156-8-15 (PMC1857697; doi:10.1186/1471-2156-8-15)
Supplement: Additional file 2 — Genotype frequencies within individual breeds. The table provided lists the genotype frequencies for each breed separately. [file 1471-2156-8-15-S2.doc]

## Additional File 2 - Genotype frequencies within individual breeds

|  | **23 bp indel** | | | | |  | **12 bp indel** | | | | |
| --- | --- | --- | --- | --- | --- | --- | --- | --- | --- | --- | --- |
|  | BSE | n | Control | n | P |  | BSE | n | Control | n | P |
| German Holstein |  | 119 |  | 80 |  |  |  | 119 |  | 80 |  |
| D/D | 0.429 | 51 | 0.425 | 34 | 0.8654 |  | 0.345 | 41 | 0.338 | 27 | 0.9429 |
| D/I | 0.504 | 60 | 0.488 | 39 |  |  | 0.529 | 63 | 0.550 | 44 |  |
| I/I | 0.067 | 8 | 0.088 | 7 |  |  | 0.126 | 15 | 0.113 | 9 |  |
| **German Fleckvieh** |  | 69 |  | 60 |  |  |  | 69 |  | 60 |  |
| D/D | 0.623 | 43 | 0.500 | 30 | 0.3461 |  | 0.522 | 36 | 0.467 | 28 | 0.1128 |
| D/I | 0.304 | 21 | 0.383 | 23 |  |  | 0.391 | 27 | 0.317 | 19 |  |
| I/I | 0.072 | 5 | 0.117 | 7 |  |  | 0.087 | 6 | 0.217 | 13 |  |
| German Brown |  | 16 |  | 41 |  |  |  | 16 |  | 41 |  |
| D/D | 0.250 | 4 | 0.146 | 6 | 0.6001 |  | 0.063 | 1 | 0.000 | 0 | 0.1613 |
| D/I | 0.563 | 9 | 0.585 | 24 |  |  | 0.438 | 7 | 0.317 | 13 |  |
| I/I | 0.188 | 3 | 0.268 | 11 |  |  | 0.500 | 8 | 0.683 | 28 |  |
| **Swiss Brown** |  | 98 |  | 103 |  |  |  | 98 |  | 103 |  |
| D/D | 0.235 | 23 | 0.184 | 19 | 0.6242 |  | 0.071 | 7 | 0.029 | 3 | 0.3861 |
| D/I | 0.357 | 35 | 0.408 | 42 |  |  | 0.378 | 37 | 0.398 | 41 |  |
| I/I | 0.408 | 40 | 0.408 | 42 |  |  | 0.551 | 54 | 0.573 | 59 |  |
| **Swiss Schwarzfleck** |  | 25 |  | 26 |  |  |  | 25 |  | 26 |  |
| D/D | 0.400 | 10 | 0.192 | 5 | 0.2251 |  | 0.280 | 7 | 0.154 | 4 | 0.3442 |
| D/I | 0.480 | 12 | 0.577 | 15 |  |  | 0.560 | 14 | 0.538 | 14 |  |
| I/I | 0.120 | 3 | 0.231 | 6 |  |  | 0.160 | 4 | 0.308 | 8 |  |
| **Swiss Simmental x Red Holstein** |  | 122 |  | 121 |  |  |  | 122 |  | 121 |  |
| D/D | 0.369 | 45 | 0.314 | 38 | 0.1425 |  | 0.238 | 29 | 0.248 | 30 | 0.7568 |
| D/I | 0.516 | 63 | 0.479 | 58 |  |  | 0.492 | 60 | 0.446 | 54 |  |
| I/I | 0.115 | 14 | 0.207 | 25 |  |  | 0.270 | 33 | 0.306 | 37 |  |
