# Supplementary material for: PRNP promoter polymorphisms are associated with BSE susceptibility in Swiss and German cattle
Source: BMC Genet. 2007 Apr 16;8:15. doi: 10.1186/1471-2156-8-15 (PMC1857697; doi:10.1186/1471-2156-8-15)
Supplement: Additional file 3 — Haplotype frequencies within individual breeds. The table provided lists the haplotype frequencies for each breed separately. [file 1471-2156-8-15-S3.doc]

**Additional File 3 - Haplotype frequencies within individual breeds**

|  | BSE | n | Control | n | *P* |
| --- | --- | --- | --- | --- | --- |
| German Holstein |  | 238 |  | 160 | 0.7481 |
| D-D | 0.605 | 144 | 0.613 | 98 |  |
| D-I | 0.076 | 18 | 0.056 | 9 |  |
| I-I | 0.319 | 76 | 0.331 | 53 |  |
| German Fleckvieh |  | 138 |  | 120 | 0.2729 |
| D-D | 0.717 | 99 | 0.625 | 75 |  |
| D-I | 0.058 | 8 | 0.067 | 8 |  |
| I-I | 0.225 | 31 | 0.308 | 37 |  |
| German Brown |  | 32 |  | 82 | 0.3261 |
| D-D | 0.281 | 9 | 0.159 | 13 |  |
| D-I | 0.250 | 8 | 0.280 | 23 |  |
| I-I | 0.469 | 15 | 0.561 | 46 |  |
| Swiss Brown |  | 196 |  | 206 | 0.6667 |
| D-D | 0.260 | 51 | 0.223 | 46 |  |
| D-I | 0.153 | 30 | 0.170 | 35 |  |
| I-I | 0.587 | 115 | 0.607 | 125 |  |
| Swiss Schwarzfleck |  | 50 |  | 52 | 0.2691 |
| D-D | 0.560 | 28 | 0.423 | 22 |  |
| D-I | 0.080 | 4 | 0.058 | 3 |  |
| I-I | 0.360 | 18 | 0.519 | 27 |  |
| Swiss Simmental x Red Holstein |  | 244 |  | 242 | 0.0484 |
| D-D | 0.480 | 117 | 0.471 | 114 |  |
| D-I | 0.148 | 36 | 0.083 | 20 |  |
| I-I | 0.373 | 91 | 0.446 | 108 |  |
